# Mismatch repair proteins play a role in ATR activation upon temozolomide treatment in *MGMT*-methylated glioblastoma

**DOI:** 10.1038/s41598-022-09614-x

**Published:** 2022-04-06

**Authors:** Sachita Ganesa, Amrita Sule, Ranjini K. Sundaram, Ranjit S. Bindra

**Affiliations:** 1 Department of Molecular Biophysics and Biochemistry, Yale University, New Haven, CT 06511 USA; 2 Department of Therapeutic Radiology, Yale University, 333 Cedar Street, New Haven, CT 06511 USA

**Keywords:** Cancer, Drug discovery

## Abstract

The methylation status of the O^6^-methylguanine methyltransferase (*MGMT*) gene promoter has been widely accepted as a prognostic biomarker for treatment with the alkylator, temozolomide (TMZ). In the absence of promoter methylation, the MGMT enzyme removes O^6^-methylguanine (O^6^-meG) lesions. In the setting of *MGMT*-promoter methylation (MGMT-), the O^6^-meG lesion activates the mismatch repair (MMR) pathway which functions to remove the damage. Our group reported that loss of MGMT expression via *MGMT* promoter silencing modulates activation of ataxia telangiectasia and RAD3 related protein (ATR) in response to TMZ treatment, which is associated with synergistic tumor-cell killing. Whether or not MMR proteins are involved in ATR activation in MGMT-cells upon alkylation damage remains poorly understood. To investigate the function of MMR in ATR activation, we created isogenic cell lines with knockdowns of the individual human MMR proteins MutS homolog 2 (MSH2), MutS homolog 6 (MSH6), MutS homolog 3 (MSH3), MutL homolog 1 (MLH1), and PMS1 homolog 2 (PMS2). Here, we demonstrate that MSH2, MSH6, MLH1 and PMS2, specifically, are involved in the activation of the ATR axis after TMZ exposure, whereas MSH3 is likely not. This study elucidates a potential mechanistic understanding of how the MMR system is involved in ATR activation by TMZ in glioblastoma cells, which is important for targeting MMR-mutated cancers.

## Introduction

The enzyme O^6^-methylguanine methyltransferase (MGMT) removes alkyl damage at the O^6^ position of guanine by transferring the alkyl adduct to itself at cysteine-145 before degradation by the ubiquitin proteolytic pathway^1–5^. The expression of MGMT is dependent upon its promoter methylation status, which has been widely used as a predictive biomarker to the sensitivity of alkylating agents in glioblastoma (GBM)^6^. It is well-established that glioblastoma patients with a methylated *MGMT*-promoter have better overall survival when treated with the alkylating agent, temozolomide (TMZ)^7^. Though there has been evidence of low MGMT expression in other tumors such as colorectal carcinomas, small-cell lung carcinomas, lymphomas, and head and neck carcinomas, TMZ treatment has been mostly limited to glioblastomas^8–10^.

TMZ is an alkylating agent that creates O^6^-methyl lesions on guanine residues of DNA. As mentioned, when MGMT is present (MGMT +), these lesions are removed and O^6^-methylguanine (O^6^meG) can return to its original form of guanine^5^. In the absence of MGMT due to promoter methylation (MGMT-), the O^6^meG lesions remain unrepaired, and these lesions mispair with thymine^11^. This O^6^meG:T mismatch will recruit the mismatch repair (MMR) pathway^12^. The MMR pathway encompasses two main heterodimers: MutSα and MutLα^13^*.* The MutSα heterodimer, which is comprised of proteins MutS homolog 2 (MSH2) and MutS homolog 6 (MSH6), is responsible for recognizing the O^6^meG:T mispair^14^. Upon recognition of the mispair, the MutLα heterodimer, which is comprised of proteins MutL homolog 1 (MLH1) and PMS1 homolog 2 (PMS2), acts as an endonuclease to nick the DNA, likely recruiting exonuclease I (EXO1) to remove the thymine lesion^15^. Then, replicative polymerases are likely responsible for replacing the mispaired lesion through base substitution^13^. A lesser studied heterodimer, MutSβ, composed of MSH2 and MutS homolog 3 (MSH3), has been shown to repair both small-loop and large-loop repair, without being involved in base substitution^13,14^. The continual replacement of thymine with itself leads to iterative futile cycling through various rounds of DNA replication, which is believed to be the cause of TMZ toxicity in the MGMT- setting^16,17^.

Work from our group and others have shown that DNA replication stress and thus ataxia telangiectasia and RAD3 related protein (ATR) activation could serve as an additional mechanism of TMZ toxicity^18^. During replication stress, replication protein A (RPA) coats single-stranded DNA preventing reannealing, which recruits ATR^19,20^. RPA is phosphorylated by ATR at the serine 33 residue, acting as a marker of replication stress and activating other DNA repair proteins^20^. In addition to phosphorylating RPA, ATR also phosphorylates protein CHEK1 (CHK1) at the serine 317 and serine 345 residues^21^. ATR activation and CHK1 phosphorylation can ultimately lead to a G_2_ arrest in response to DNA damage^19–21^.

Though the MMR pathway is vital in maintaining the integrity of our DNA, its specific role in removing TMZ-induced lesions remains poorly understood^22^. Studies have shown that alkylation damage can cause MMR proteins to recruit other DNA repair proteins such as ATR, though this work is the subject of contentious debate^23–25^. There has been evidence that the MutSα and MutLα heterodimers recruit ATR, though the specifi*c* mechanism of MMR-induced ATR activation upon alkylation damage remains unclear^23–25^. Here, we report the role of 5 individual MMR proteins (MSH2, MSH6, MLH1, PMS2, and MSH3) in ATR activation upon TMZ treatment. We created and tested isogenic cell lines by short hairpin RNA (shRNA) silencing of all the MMR proteins individually in both the MGMT- and MGMT + setting. Mechanistically, we found that the knockdown of some MMR proteins leads to cell cycle irregularities, elevated levels of replication stress, and increased double strand breaks upon TMZ damage. We also observed that MMR-deficiency abrogates the observed synergy between TMZ and ATR inhibitors. These data reveal that the MMR pathway plays a key role in activating ATR for DNA damage, and perhaps MMR-deficiencies can be used as a biomarker for novel cancer therapies.

## Results

### Knockdown of one MMR protein affects its heterodimeric stability

**Figure 1:**
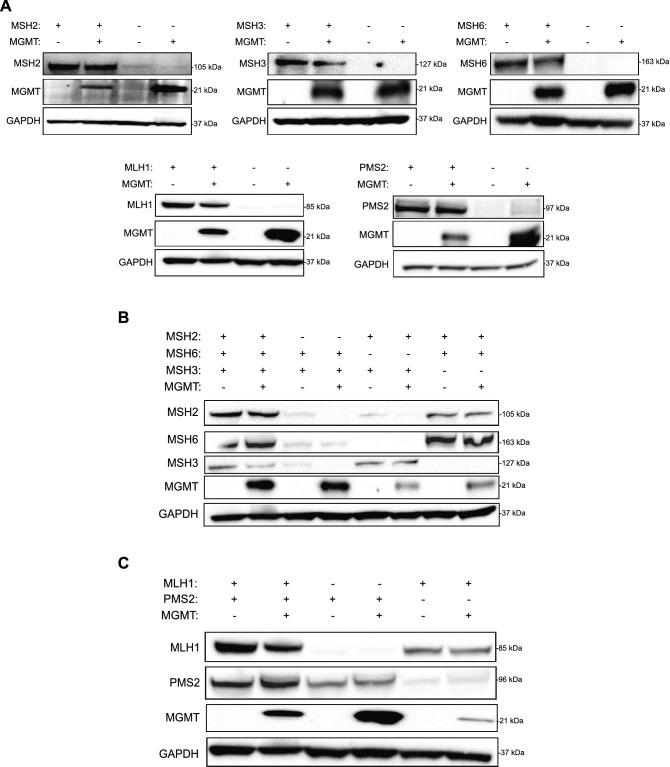
MMR knockdown of one protein affects stability of its heterodimeric partner. (**A**) Western blot showing shRNA MMR knockdown in LN229 cells, in both the MGMT- and MGMT + cell lines. (**B**) Levels of MSH2, MSH6, and MSH3 were assessed in shMSH2, shMSH6, and shMSH3 MGMT- and MGMT + cells. The membrane was stripped and reprobed for MSH6, and then MSH3 after the initial MSH2 exposure. (**C**) Levels of MLH1 and PMS2 were assessed by western blotting in shMLH1 and shPMS2 cells. The membrane was stripped and reprobed for PMS2 after initial MLH1 exposure. Full length blots are shown in the supplemental.

First, we sought to create a cell line model system to study how the loss of MMR proteins affects TMZ-mediated ATR activation. We knocked down MSH2, MSH3, MSH6, MLH1 and PMS2 individually in LN229 glioblastoma cells using the GIPZ lentiviral shRNA knockdown system. Importantly, we created these MMR knockdowns to be in both an MGMT- (MGMT-deficient) and MGMT + (MGMT-proficient) cell line. After screening for monoclonal cell populations of each cell line, we confirmed the knockdown of individual MMR proteins (Fig. 1A).

MSH2 exists in a heterodimeric complex with MSH6 (MutSα complex) or MSH3 (MutSβ complex), as does MLH1 and PMS2 (MutLα complex). We wanted to begin by testing whether MMR protein knockdown would affect the stability of its heterodimeric partner, as has been done previously in other contexts but is unknown in these LN229 glioblastoma cells^26–28^. To this end, we ran a western blot on whole cell lysates of all shMMR cell lines and probed for MSH2, MSH6, and MSH3 along with MLH1 and PMS2. We observed reduced levels of MSH2 protein in the shMSH6 cells, like the levels seen in the shMSH2 cells. Likewise, we observed reduced levels of MSH6 in shMSH2 cells. There was slight reduction of MSH2 protein in the shMSH3 cells as well, but not vice versa (Fig. 1B). These data suggest that the stability of the MSH2-MSH6 complex is lost when one of the heterodimeric components is knocked down, though the MSH2-MSH3 heterodimer remains somewhat stable. Additionally, knocking down MLH1 slightly reduced the expression of PMS2 and vice versa (Fig. 1C), which could possibly disrupt the heterodimer. From literature, we know that MMR proteins play an important role in determining treatment outcomes^29–31^. Thus, our cell line models will be used to assess and elucidate the specific roles of these individual MMR proteins in TMZ-mediated ATR activation.

### Functional MMR is required for synergy between TMZ and ATR inhibitors

Previously published work from our lab detailed that MGMT status affects the interaction between TMZ and ATR inhibitors^18^. We first recapitulated the findings of this paper, showing sensitivity of MGMT- cells to TMZ in both short-term cell viability assays and clonogenic survival assays (Supplementary Fig. S1A-B). We performed CHK1 pulldown on LN229 cells treated with low to high doses of TMZ and assessed phosphorylation on CHK1 at various time points. In line with what we had observed previously, we saw earlier activation of ATR, seen as increased phosphorylation on CHK1 in MGMT- cells relative to MGMT + cells (Supplementary Fig. S1C-D). We have shown that MGMT status affects ATR activation post TMZ treatment.

Before moving forward, we wanted to validate whether the MGMT-dependent synergy observed is specific to the ATR kinase. We therefore assessed synergy between inhibitors of other PI3-kinases like ATM (AZD0156) and DNA-PK (AZD7648) in combination with TMZ in both MGMT- and MGMT + cells. We showed that there is some synergy between AZD0156 or AZD7648 and TMZ and in both MGMT- and MGMT + cells (Supplementary Fig. S2A-B). This strengthened our observation that MGMT status plays a unique role in affecting synergy between TMZ and ATR inhibitors only, and not other kinase inhibitors. Having confirmed that synergy between TMZ and ATR inhibitors depends on MGMT status, we wanted to understand whether and how MMR could play a role in this interaction.

It has been shown that certain MMR deficient cancers do not respond to treatment with TMZ, but it is not yet known which MMR proteins are involved in conferring TMZ resistance^30,32–34^. Thus, we sought to investigate each MMR protein individually for its sensitivity to TMZ and ATR inhibitors. We performed short-term cell viability assays with LN229 MGMT- cells and the LN229 MGMT- MMR knockdown cells to assess their sensitivity to TMZ and the ATR inhibitor BAY-1895344. The LN229 MGMT- shMSH2, shMSH6, shPMS2, and shMLH1 cells were all resistant to treatment with TMZ compared to the LN229 MGMT- MMR-proficient cells and the shMSH3 cells, which were both sensitive to TMZ treatment (Supplementary Fig. S3A). We performed short-term cell viability assays using BAY-1895344 on the MMR-deficient cell lines and observed that they were all sensitive to this treatment like the LN229 MGMT- MMR-proficient cells and observed similar level of sensitivity among the cells lines with the shMSH2 cells being the most sensitive (Supplementary Fig. S3B).

**Figure 2:**
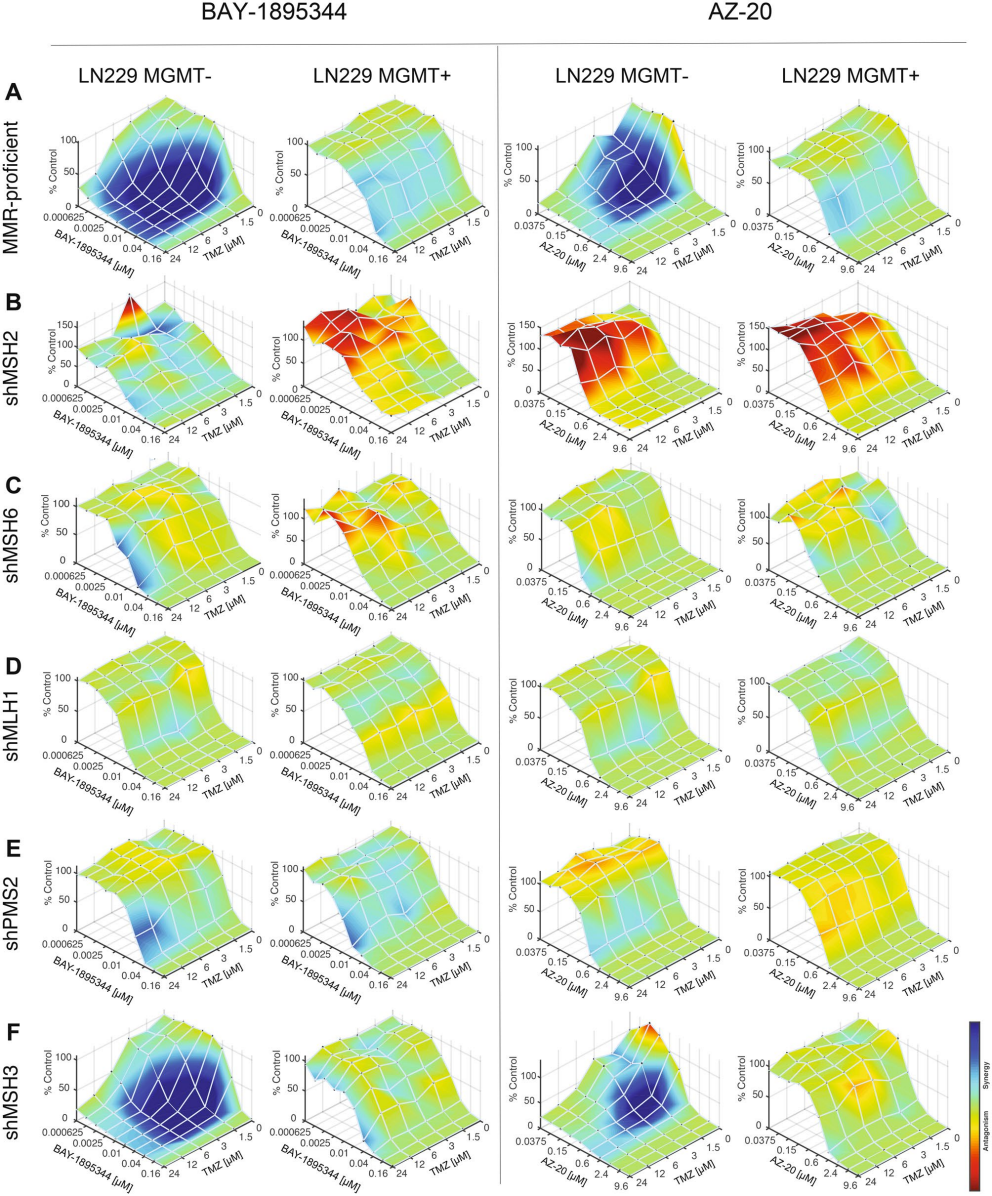
MMR proteins are required for synergy between TMZ and two structurally unique and ATR inhibitors, BAY-1895344 and AZ-20. Synergy assays were performed between TMZ and BAY-1895344 or AZ-20 in the newly created MMR-deficient LN229 isogenic cell lines in triplicate plates before being analyzed by Combenefit (*n* = 3). Cells were treated for 6 days with the combination of inhibitors. (**A**) MGMT-cells that are MMR-proficient exhibit exquisite synergy upon treatment with TMZ and BAY-1895344 or AZ-20, but there is a lack of synergy in the MGMT + cells. (**B**) shMSH2, (**C**) shMSH6, (**D**) shMLH1, and (**E**) shPMS2 cells do not display synergy for the combination of TMZ and BAY-1895344 or AZ-20 whereas (**F**) shMSH3 MGMT- cells do show synergy. This indicates that the knockdown of MSH2, MSH6, MLH1, and PMS2 affect synergy between TMZ and ATR inhibitors, suggesting a role for these proteins in ATR signaling.

As detailed in Jackson et al., 2019, TMZ sensitizes MGMT- tumor cells to ATR inhibitors; however, the extent to which MMR plays a role in modulating this synergistic interaction is unknown. We tested if MMR-deficient TMZ-resistant cells would be sensitized to the combination of TMZ and ATR inhibitors, BAY-1895344 and AZ-20. As seen previously, we observed exquisite synergy in LN229 MGMT- cells when treated with TMZ and BAY-1895344 or AZ-20 which was not seen in LN229 MGMT + cells (Fig. 2A). However, cells with the MMR deficiencies of MSH2, MSH6, MLH1, and PMS2 did not synergize with TMZ and BAY-1895344 or AZ-20 (Fig. 2B-E). Interestingly, we observed synergy between TMZ and BAY-1895344 or AZ-20 in the MSH3-deficient cells (Fig. 2F), suggesting that the MSH2-MSH6 and MLH1-PMS2 heterodimers could be responsible for attending to TMZ-induced mismatched lesions, as opposed to the MSH2-MSH3 heterodimer. Cells that were MMR-deficient and MGMT + also did not exhibit synergy, signifying that MGMT-methylation status and MMR status are equally important for synergistic interactions between TMZ and ATR inhibitors.

**Figure 3:**
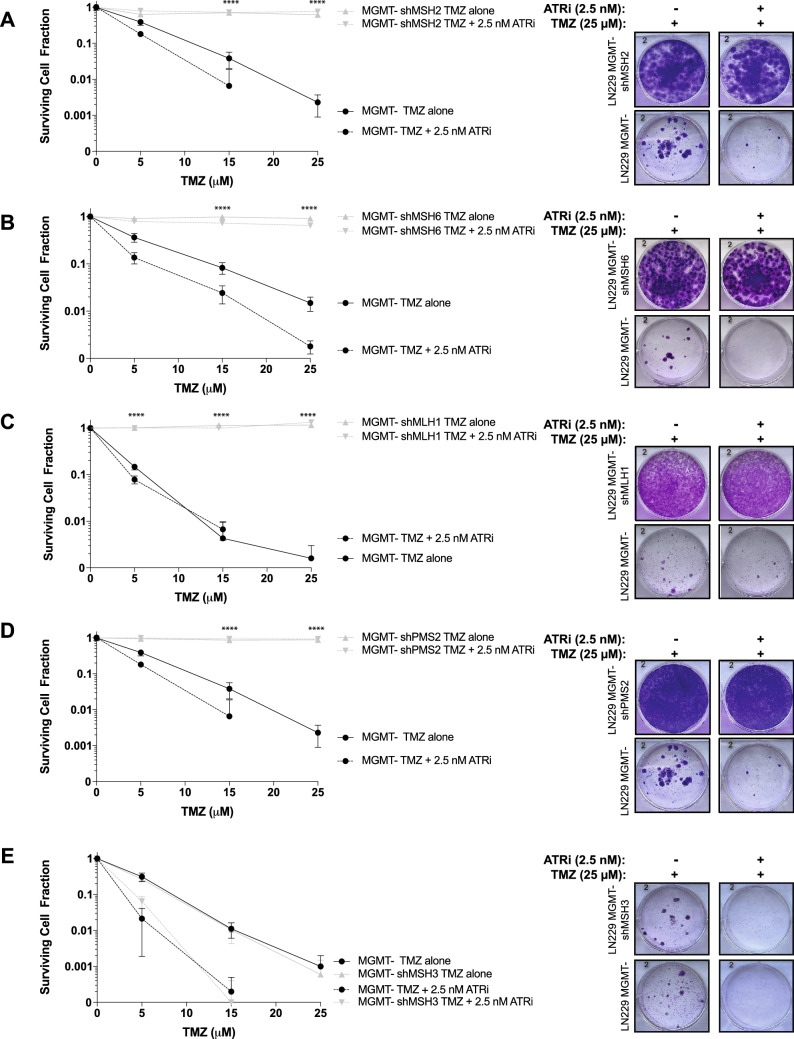
MMR-deficient cells are resistant to the combination of TMZ and ATR inhibitor in clonogenic survival assay. LN229 cells were pre-treated with 12.5 μM of TMZ for 72 h before counting and seeding these cells into new plates with fresh media. Cells were seeded at various dilutions in triplicate (*n* = *3*) before adding BAY-1895344 at a constant concentration of 2.5 nM for 14 days. Plates remained undisturbed in the incubator for 14 days before staining and counting colonies. (**A**) shMSH2, (**B**) shMSH6, (**C**) shMLH1, and (**D**) shPMS2 cells are resistant to TMZ and the combination with BAY-1895344. (**E**) shMSH3 cells are sensitive to TMZ and BAY-1895344. In all graphs, the solid black line indicates the wild-type LN229 MMR-proficient MGMT- cells treated with TMZ and the dashed black line indicates the same cells treated with TMZ and ATR inhibitor BAY-1895344. The solid gray line represents the shRNA MMR knockdown cell line treated with TMZ alone, and the dashed gray line represents the shRNA MMR knockdown cell line treated in combination with TMZ and BAY-1895344. Standard deviation was calculated from the triplicate values of colonies counted. Unpaired *t-*test was performed using GraphPad PRISM between LN229 MGMT- wild-type cells (dashed black line) and the shMMR cells (dashed grey line) for the TMZ + BAY-1895344 combination (****p < 0.0001).

We performed clonogenic survival assays using TMZ and BAY-1895344. The LN229 MGMT- cells showed sensitivity to TMZ alone, and we saw increased sensitivity to the combination of TMZ and BAY-1895344 (Fig. 3). The LN229 MGMT- shMSH2, shMSH6, shMLH1, and shMSH6 cells were all resistant to treatment with TMZ, and furthermore, did not respond to the combination treatment of TMZ with BAY-1895344 (Fig. 3A-D). As expected from the synergy assays, the LN229 MGMT- shMSH3 cells were sensitive to TMZ and to the combination of both drug treatments (Fig. 3E).

We sought to recapitulate the MMR deficiency phenotype observed in the LN229 MGMT- cells using another glioma cell line. We tested the combination of TMZ and BAY-1895344 or AZ-20 in an isogenic glioma cell line model using U251 wild-type cells and U251 shMSH2 cells, which are both MGMT- (Supplementary Fig. S4A). As we saw with the LN229 MGMT- shMSH2 cells, the U251 shMSH2 cells were also resistant to TMZ as a monotherapy (Supplementary Fig. S4B). Additionally, they did not exhibit synergy when treated with TMZ and BAY-1895344 or AZ-20, and were resistant to TMZ and BAY-1895344 in a clonogenic survival assay (Supplementary Fig. S4C-E). Collectively, these data suggest that MMR is required for the synergistic interaction between TMZ and ATR inhibitors, and that MMR could be involved in the induction of TMZ induced-ATR repair.

### MMR is implicated in the ATR/CHK1 signaling axis

**Figure 4:**
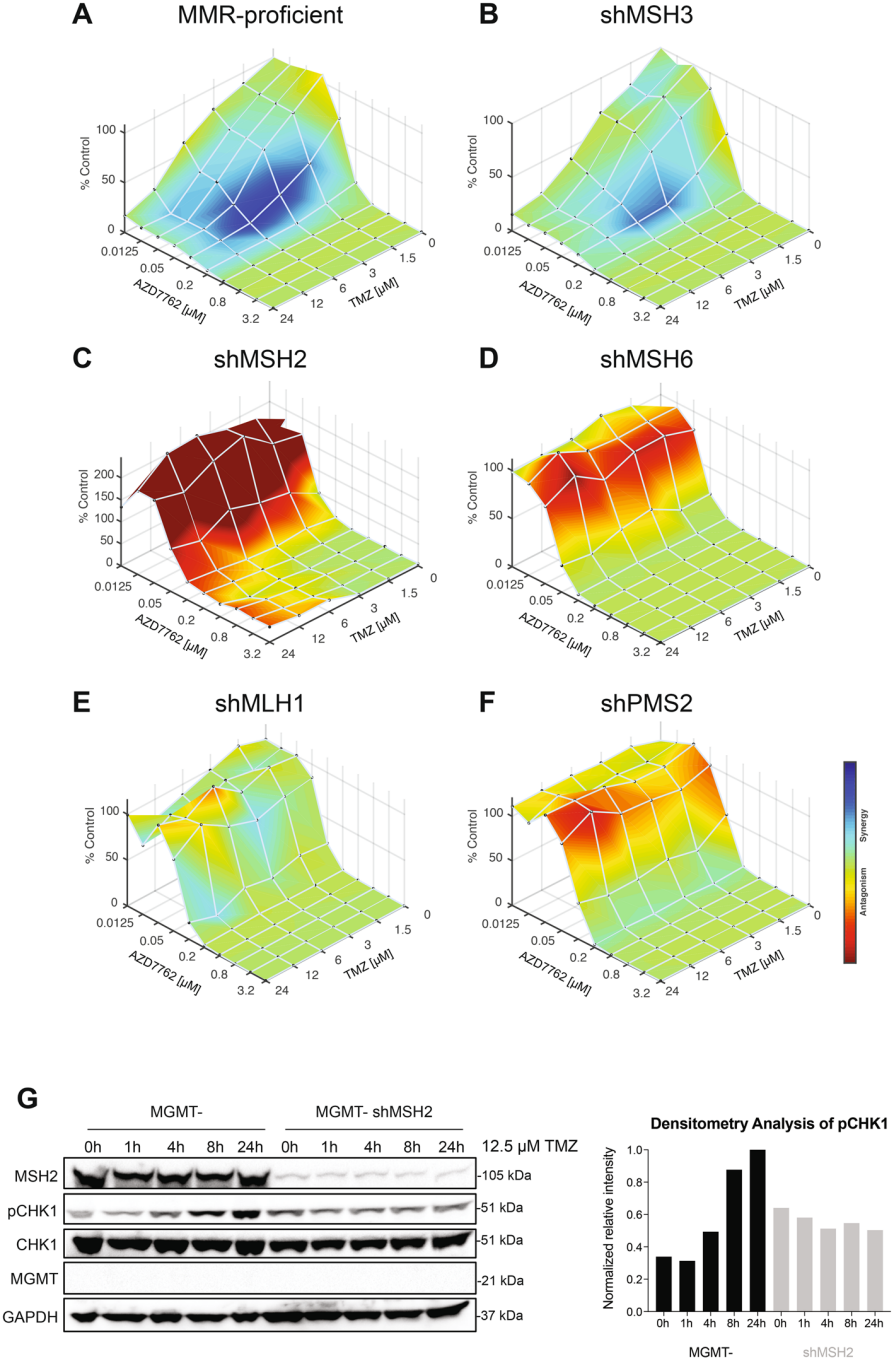
MMR is likely implicated in the entire ATR/CHK1 signaling axis. Synergy assays were performed between TMZ and AZD7762 in the newly created MMR-deficient LN229 isogenic cell lines in triplicate plates before being analyzed by Combenefit. Cells were treated for 6 days with the combination of inhibitors. (**A**) MGMT- cells that are MMR-proficient and (**B**) shMSH3 MGMT- cells exhibit some synergy upon treatment with TMZ and AZD7762. (**C**) shMSH2, (**D**) shMSH6, (**E**) shMLH1, and (**F**) shPMS2 MGMT- cells do not display synergy for the combination of TMZ and AZD7762. This indicates that the knockdown of MSH2, MSH6, MLH1, and PMS2 adversely affects synergy between TMZ and AZD7762, suggesting a role for these proteins in ATR-CHK1 signaling. (**G**) Western blot of MGMT- and shMSH2 MGMT- cells showing the kinetics of ATR activation through pCHK1 levels after 12.5 µM of TMZ treatment for the time course indicated. From the densitometry analysis of this blot, there is a robust increase in pCHK1 levels over time in the MGMT- MMR-proficient cells, but not in the shMSH2 cells, suggesting that MSH2 is required for ATR activation and signaling to CHK1. The densitometry analysis is based upon the pCHK1 levels from this representative western blot. Full length blots are shown in the supplemental.

Next, we wanted to test the extent to which MMR is required in the ATR/CHK1 signaling axis. We started by testing if MMR-deficient cells would be sensitized to the combination of TMZ and a CHK1/2 inhibitor, AZD7762. We saw very little synergy in the MMR-proficient cells when treated with TMZ and AZD7762, indicating that MGMT-status is mostly specific to sensitizing TMZ to ATR inhibitors (Fig. 4A). We also saw this slight synergy in the shMSH3 cells (Fig. 4B). However, cells with the MMR deficiencies of MSH2, MSH6, MLH1, and PMS2 exhibited a lack of synergy with TMZ and AZD7762 (Fig. 4C-F). This suggested that the MMR proteins MSH2, MSH6, MLH1, and PMS2 are involved in the entire ATR/CHK1 signaling axis as the knockdown of these proteins causes antagonism when treated with TMZ and AZD7762. Further, we wanted to test whether MMR loss would affect the kinetics of ATR activation through pCHK1 signal. We observed that there was a lack of robust ATR activation seen through the consistent levels of pCHK1 over time in the LN229 MGMT- shMSH2 cells treated with TMZ (Fig. 4G). This contrasted with the increased pCHK1 signal and thus ATR activation over time in the LN229 MGMT- cells.

### Mismatch repair deficiency affects cell cycle and increases DNA replication stress

**Figure 5:**
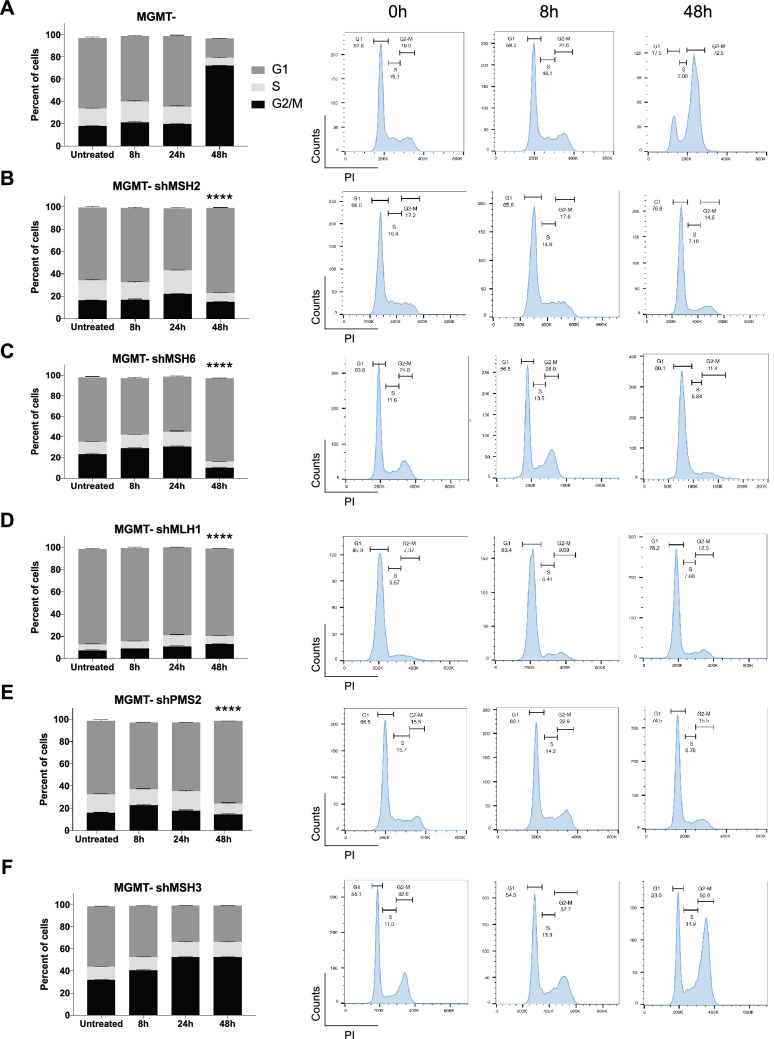
MMR is required for ATR-activated G_2_-M arrest. Cells were treated with 12.5 µM of TMZ for the timepoints indicated before being harvested for propidium-iodide staining in a 96-well plate format. Data was acquired on a CytoFLEX Flow Cytometer and analyzed with FlowJo software. Data are presented as mean ± SEM (*n* = 3). (**A**) TMZ causes G_2_/M arrest as seen in the MMR-proficient cells over 48 h of treatment, which is a sign of increased ATR activation. However, the (**B**) shMSH2, (**C**) shMSH6, (**D**) shMLH1, and (**E**) shPMS2 cells are not arrested in G_2_/M and rather cycle normally in G_1_ as the untreated condition, even after 48 h of TMZ treatment. (**F**) shMSH3 cells are arrested in G_2_/M after 48 h of temozolomide treatment like the wild-type LN229 MGMT- cells. This suggests that MSH2, MSH6, MLH1, and PMS2 are required for proper ATR signaling and cell cycling compared to MSH3. Statistical testing was performed using GraphPad PRISM. Where indicated, ****p < 0.0001 comparing the 48 h timepoint of MGMT- G_2_/M with the 48 h timepoint of the other cell lines in a one-way ANOVA with multiple comparisons.

We went on to probe the mechanism for distinct responses of MMR protein deficiencies to TMZ-mediated damage. As described previously, MGMT- cells treated with TMZ undergo G_2_/M arrest in the cell cycle, which is thought to be due to ATR activation and CHK1 phosphorylation^18,20,21^. We sought to understand whether MMR deficiency would affect cell cycle progression and phase distribution. After treating cells with TMZ over the course of 48 h, we stained the cells with propidium-iodide (PI) for cell cycle analysis using flow cytometry. We observed an increase in G_2_/M arrest in the MGMT- cells (Fig. 5A) after 48 h of TMZ treatment. The MGMT-shMSH2 cells did not exhibit G_2_/M arrest, suggesting that the MSH2 loss leads to reduced ATR activation in these cells (Fig. 5B). The MGMT-shMSH6 and shPMS2 and shMLH1 cells also did not appear to be arrested in G_2_/M, but rather remained mostly in G_1_ even after 48 h of TMZ treatment (Fig. 5C-E). This indicated that the knockdown of these MMR proteins prevents or blocks ATR activation, leading to an abrogation of G_2_/M arrest. Said otherwise, these MMR proteins are required for ATR signaling and G_2_/M arrest. The MGMT-shMSH3 cells were arrested in G_2_/M after 48 h of TMZ treatment like the MGMT- cells, suggesting that MSH3 is not required for ATR signaling (Fig. 5F).

**Figure 6:**
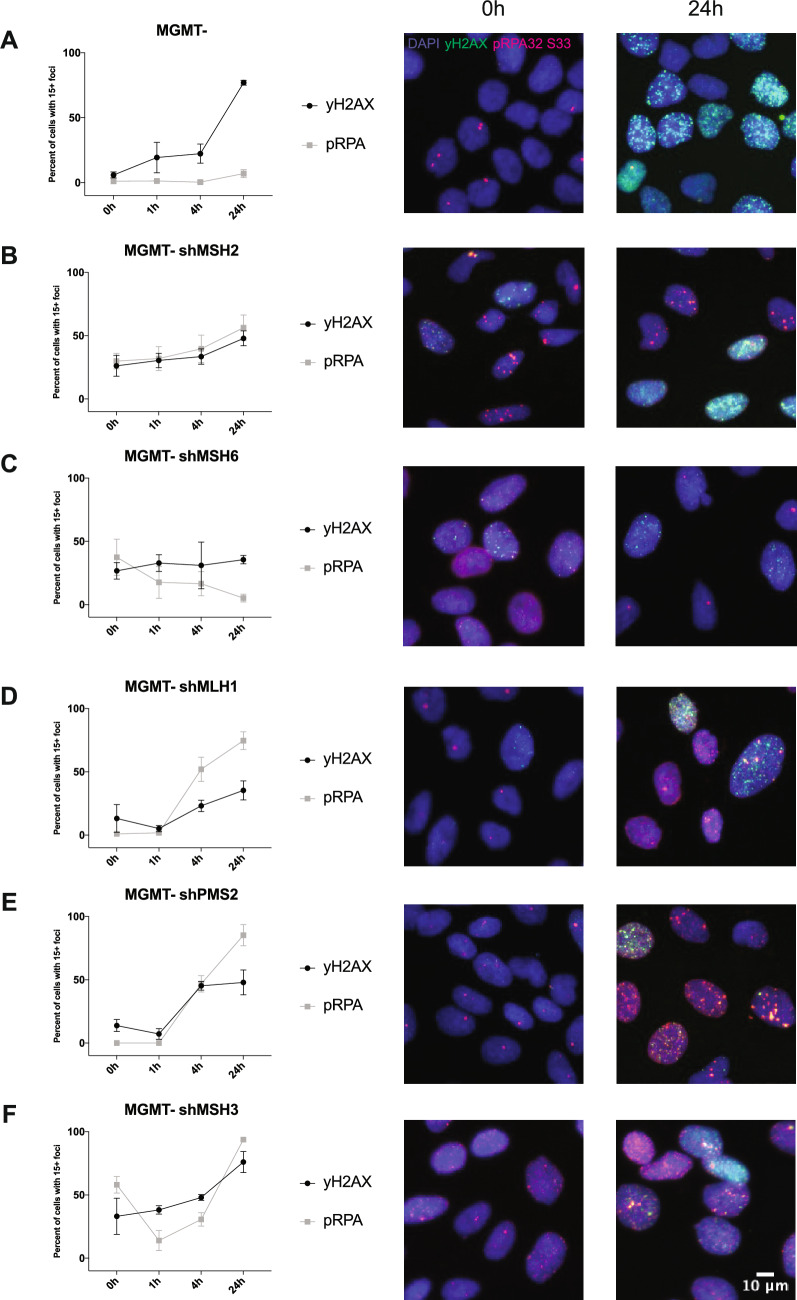
MMR-deficiency affects DNA repair with increased replication stress and double strand breaks. Cells were seeded in triplicate and subsequently treated with 12.5 µM of TMZ over the course of 24 h at the indicated timepoints before being fixed and stained with pRPA32 S33, γH2AX, and Hoescht nuclear stain. Cells were imaged on a Keyence BZ-X800 and analyzed using Focinator software. (**A**) LN229 MGMT-, (**B**) MGMT- shMSH2, (**C**) MGMT- shMSH6, (**D**) MGMT- shMLH1, (**E**) MGMT- shPMS2, and (**F**) MGMT- shMSH3 cells show differences in foci numbers and trends. MGMT- cells show increased γH2AX over time, indicating more double strand breaks but low levels of pRPA, suggesting lack of replication stress. The MMR-deficient cells have varying levels of each both pRPA and γH2AX, suggesting a unique role for each individual MMR protein in repairing TMZ-induced lesions. Data is shown in triplicate (*n* > 500 cells) and is represented as mean ± SD.

Given that we observed dysregulation of cell cycle in MMR-deficient cells, we wanted to know if these cells had increased levels of replication stress. This could provide us with insight into why the TMZ/ATR inhibitor synergy is abrogated in several MMR-deficient cell lines. ATR phosphorylates RPA at serine 33, which serves as a sign of replication stress and can be observed through immunofluorescence^20,35^. We also assessed the MMR-deficient cells for increased double-strand breaks over time with TMZ treatment, seen through γH2AX immunofluorescence. In the MGMT- cells, we saw low and steady levels of pRPA foci over time, suggesting that there was relatively less replication stress upon TMZ treatment (Fig. 6A). However, we saw an increase in γH2AX over time, suggesting increased double strand breaks, consistent with previously published data^18^ (Fig. 6A). This alluded to the futile cycling model, where overtime the TMZ-induced damage with functional MMR leads to single-strand DNA breaks and ultimately double-strand DNA breaks.

The MGMT- shMSH2 cells showed increases in both pRPA and γH2AX foci over time, though there was only a slight increase (Fig. 6B). This indicated that there is a baseline elevated level of replication stress and double-strand breaks in the MSH2 knockdown cells which stayed consistent over time compared to the MGMT- cells. MSH6, which partners with MSH2 in the MutSα heterodimer, also showed baseline elevated levels of pRPA and γH2AX. The pRPA levels decreased over 24 h, indicating some resolution of replication stress that may have occurred (Fig. 6C). This decrease in pRPA levels was consistent with the cell cycle data showing that these cells were back to normal cell cycling. In contrast, MSH3, which also partners with MSH2, saw increasing levels of pRPA and γH2AX, indicating higher baseline levels of replication stress that increased over time, likely leading to more double strand breaks, once again consistent with the cell cycle data (Fig. 6F). MLH1 and PMS2 which comprise the MutLα heterodimer showed increased pRPA and γH2AX foci over time over 24 h of TMZ treatment (Fig. 6D-E). This suggests that replication stress is increasing in tandem with double strand breaks.

Together, this flow cytometry data suggests that mechanistically, MSH2, MSH6, MLH1, and PMS2 play a role in ATR signaling upon TMZ-induced lesions but not MSH3. Further, the immunofluorescence data potentially elucidates a unique role for each individual MMR protein based upon the varying levels of replication stress and double strand breaks in each MMR knockdown cell line.

## Discussion

In this study, we report distinct roles for MMR proteins upon TMZ treatment. We created isogenic cell lines containing MMR knockdowns in both the MGMT- and MGMT + backgrounds. Our data from synergy and clonogenic assays with TMZ and ATR inhibitor combination indicated that apart from MSH3, all the other MMR proteins are likely involved in ATR signaling. We also probed further to elucidate the mechanism of MMR-induced ATR activation. Our group has previously shown that TMZ can sensitize MGMT-deficient tumors to ATR inhibitors^18^, but here we report that MMR is required for this combination of inhibitors to be effective. Emerging data shows that various tumors harbor mutations in the MMR proteins, such as mutations in MSH2 or MSH6 in recurrent GBM, or MLH1 in colorectal cancer^29,32,36^. Understanding the mechanism of the MMR pathway can provide insight into whether these MMR-mutated cancers may benefit from certain treatment strategies.

To our knowledge, this is the first study to report that MMR-deficient tumor cells did not respond to the combination of TMZ and ATR inhibitors. Overall, this work provides us with a novel understanding of the role of specific MMR proteins, which can be used as a biomarker in selecting the correct treatment regimen for patients to enhance the therapeutic index. As observed, there was exquisite synergy between TMZ and ATR inhibitors BAY-1895344 and AZ-20 in the MGMT- MMR-proficient cells. Our work here showed that this synergy likely depends on the functional MMR proteins MSH2, MSH6, MLH1, and PMS2. It is believed that upon TMZ damage, MMR proteins are recruited to remove the thymine lesion resulting in futile cycling; furthermore, it has also been shown that TMZ causes increased replication stress leading to ATR activation^18^. We hypothesize that treatment of cells with TMZ is causing replication stress activating ATR, which, when treated in combination with an ATR inhibitor, leads to increased cell death, or synergy. We showed here that MMR proteins are likely involved in activating ATR upon TMZ, and thus the knockdown of MMR proteins would prevent ATR activation. This knockdown would have little effect on cell death when treating with an ATR inhibitor, which is likely why we saw an abrogation of synergy in MMR-deficient cells.

Though we showed destabilization of MSH6 upon MSH2-loss, the MSH6-deficient cells showed differences in pRPA and γH2AX levels versus the MSH2-cells, indicating a unique role for each protein in the dimer separately. Unlike the MSH2-MSH6 heterodimer, MSH2-MSH3 is known for repairing larger lesions, which is a possibility for why we see an increase in γH2AX foci over time and increased G_2_/M arrest in shMSH3 cells post TMZ treatment. The MSH6-deficient cells also showed decreasing pRPA levels over time, indicating a mitigation of replication stress, which could explain how the cells seemed to be cycling normally after 48 h of TMZ treatment. Given that the shMLH1 and shPMS2 cells cycle normally like the shMSH2 and shMSH6 cells but exhibited increased pRPA and γH2AX over time, we can speculate that these proteins may use different pathway regulators for repairing damage. Further, in the MGMT- MMR-proficient cells, we saw low and steady levels of pRPA foci over time, which contrasts with the data showing increasing pCHK1 levels over time upon TMZ treatment. This suggests that ATR activation does not necessarily indicate equal phosphorylation levels of its downstream targets RPA and CHK1, which has been seen prior^37^. Though further work is required to draw concrete mechanistic conclusions between replication stress, MMR, and ATR activation, our study provides evidence for the individual roles of MMR proteins in ATR activation upon TMZ treatment in *MGMT-*promoter methylated cancer cells. Our data suggest that certain MMR proteins are required for ATR signaling, and that each distinct MMR protein may serve a unique function.

In this manuscript, we have sought to systemically characterize the relative impact of unique MMR mutations in the setting of MGMT-proficient and -deficient glioma cells. To this end, we have created and validated isogenic glioma cell models to rule out any potential confounders associated with co-occurring mutations that can arise when comparing unique neurosphere models. Our study is focused on DNA repair genes and their role in the response to DNA damaging agents and DNA repair inhibitors, which is supported by numerous previous publications that have used the LN229 and U251 cell line models to demonstrate mechanisms of TMZ resistance. We acknowledge that the LN229 cells used in the majority of the work presented here have a mutation in TP53. Though 75% percent of TP53 mutations co-occur with MGMT-promoter methylation in glioblastomas, we did not consider the effects of this mutated protein specifically^38^. However, we acknowledge the significance of using these models, especially when performing in vivo studies. Additional work is ongoing to introduce MMR and MGMT knockouts in neurosphere models, and to create isogenic wild-type vs. TP53 mutant lines, which will allow us to further validate our findings in more clinically relevant models in the future.

Previously published reports have indicated that MMR is not required for ATR signaling upon alkylation damage^24,40^. One of these studies claims that low doses of TMZ do not affect MMR-induced ATR activation^40^. Here we have demonstrated that low concentrations (at the IC_50_ of around 10 µM) of TMZ can still affect ATR activation, which depends on individual MMR proteins upon TMZ-induced alkylation damage (Fig. 1B). While we did not investigate bifunctional alkylators in this study, we did thoroughly examine the role of MMR induction upon TMZ, which is a monofunctional alkylator. We were able to conclude that the monofunctional alkylating agent TMZ induces the MMR pathway for ATR activation, which can be generalized to other monofunctional alkylators such as MNNG, published previously^23,41^. Our group has previously published minimal therapeutic index of non-TMZ alkylators using *MGMT*-methylation as a marker for treatment, which is why we focused our efforts on TMZ^18^. Taken together, this work demonstrates that lesions caused by monofunctional alkylators require intact MMR pathway for activating ATR.

The MMR knockdown cell lines we created could serve to screen various combinations of treatments. For example, lomustine is a bifunctional alkylator used to treat MGMT + glioblastomas^42^. Our data here showed that the MMR-deficient MGMT- cells responded to drug treatment similarly to the MGMT + cells, so perhaps the lomustine and ATR inhibitor combination could sensitize the MMR-deficient cells. Another interesting combination therapy includes ATR inhibitors and poly (ADP-ribose) polymerase (PARP) inhibitors. PARP plays a major role in the DNA repair pathway, focusing on single-strand break repair such as in nucleotide or base excision repair. It could be interesting to mechanistically understand the role of MMR and ATR in PARP signaling by treating with PARP inhibitors and ATR inhibitors. Recently published work from our laboratory showed that PARP and ATR inhibitors synergize in the common glioblastoma mutation in isocitrate dehydrogenase 1/2 (IDH1/2), so perhaps MMR-deficiencies may also benefit from this combination^43^. Ongoing work in our laboratory continues to investigate the role of these various therapeutics in combination with ATR inhibitors in MMR-deficient cells. Other experiments are ongoing in our laboratory to further characterize MMR-deficiencies in other molecular pathways in addition to ATR signaling. These studies could provide new therapies for MMR-deficient glioblastoma patients.

## Materials and methods

### Cell culture

Human glioblastoma LN229 MGMT- and MGMT + cell lines were obtained from Bernd Kaina (Johannes Gutenberg University Mainz, Mainz, Germany). U251 cells were purchased from Horizon. All cells were confirmed negative of *Mycoplasma* using qPCR. Cells were cultured in Dulbecco’s Modified Eagle Medium (DMEM) with 10% fetal bovine serum (FBS).

### Mismatch repair knockdown cell line creation

The pGIPZ™ shRNA lentiviral vectors for mismatch repair proteins MSH2, MSH3, MSH6, MLH1 and PSM2 were purchased as glycerol stocks from Horizon Dharmacon™. Nontargeting GIPZ lentiviral shRNA particles were purchased from Horizon Dharmacon™. From the glycerol stock, the shRNA plasmids were transformed into competent cells and bacterial cultures were midiprepped (Plasmid Plus Midi Kit, #12,943 from Qiagen) before being confirmed by restriction digest with SacII. To generate lentiviral particles, HEK293T cells were transduced with the shRNA lentiviral DNA prepared from the midiprep, a packaging plasmid (psPAX2, 12,260 from Addgene), and an envelope plasmid (pCMV-VSV-G, 8454 from Addgene) using Lipofectamine™ 3000 Transfection Reagent (Invitrogen, L3000001). Viral particles were harvested from the cell media after 48 h. To create the shMMR cell lines, LN229 MGMT- and MGMT + cells were infected with the shRNA lentiviral media and 8 µg/mL of polybrene. 48 h later, cells were selected with 1 µg/mL of puromycin for 3–4 days before use. Cells were harvested as a polyclonal population and confirmed of the protein knockdown with western blotting. After confirmation of knockdown in the polyclonal population, a single cell limiting dilution was performed to create monoclonal knockdown cell populations, which were then confirmed by western blotting.

### Drug compounds

The following drug compounds were purchased from SelleckChem. Compounds were resuspended in DMSO, aliquoted, and stored at −20C: temozolomide (S1237), AZ-20 (S7050), BAY-1895344 (S9864), AZD7762 (S1532), AZD7648 (S8843), and AZD0156 (S8375).

### Western blot and immunoprecipitation

Cells were treated with drug where indicated, trypsinized with 0.05% Trypsin–EDTA (Gibco) and pelleted for use immediately or placed at –80C for long term storage. Cells were lysed on ice with 1X RIPA buffer (Cell Signaling Technology, 9806S) with 1X Halt™ Protease and Phosphatase Inhibitor Single-Use Cocktail (ThermoFisher Scientific, 78,442). The lysed cell pellets remained on ice for 30 min while vortexing every 5 min for 10 s. Lysed cell pellets were centrifuged for 10 m at 4C. The lysates were kept on ice and quantified using the Bradford protein assay, while the pellets were discarded.

For immunoprecipitation (IP), cells were lysed in lysis buffer (50 mM HEPES, 250 mM NaCl, 1% NP-40, 5 mM EDTA, and 1X Halt Protease and Phosphatase Inhibitor Single-Use Cocktail cocktail). The lysed cell pellets were sonicated 5 s on/10 s off for 15 s at 100% and then centrifuged for 25 m at 4C. The lysates were quantified using the Bradford protein assay and pellets were discarded. Equal concentrations of protein lysates (> 1 mg protein) were bound to 0.5 mg of magnetic Protein G Dynabeads (Invitrogen, 10003D) along with 1.5 µg of CHK1 antibody and rotated overnight at 4C. The following day, beads were collected with a magnetic rack and washed once with wash buffer (50 mM HEPES, 250 mM NaCl, 0.1% NP-40, 5 mM EDTA, and 1X Halt cocktail), once with high-salt wash buffer (50 mM Tris pH 7.5, 1 M NaCl, 1% Triton X-100, 1 mM DTT, 10% glycerol, and 1X Halt cocktail), and once more with wash buffer, while carefully removing the buffer in between steps on the magnetic rack. Beads were resuspended in 2X Laemmli in wash buffer before boiling for 5 m at 70C to elute the protein.

For both western blot and IP, proteins were separated using NuPAGE™ 4 to 12%, Bis–Tris gels with NuPAGE™ 1X MOPS SDS running buffer and transferred using the tank transfer method to a PVDF membrane at 90 V for 90 min at 4C. After 1 h of blocking in 5% BSA or 5% non-fat dry milk in 1X Tris Buffered Saline with 1% Tween-20 (TBS-T), membranes were incubated with primary antibody diluted 1:1000 in either 5% BSA or 5% non-fat dry milk overnight at 4C. The following primary antibodies were used from Cell Signaling Technology pCHK1 S317 (#12302), CHK1 (#2360), MGMT (#2739), MSH2 (#2850), and MLH1 (#4256). Other antibodies used include PMS2 (ProteinTech, #66075–1-Ig), MSH3 (BD Biosciences, 611,390), MSH6 (BD Biosciences, 610,918), and GAPDH (ProteinTech, HRP-60004). The following day, membranes were washed 3 times for 5 min in 1X TBS-T before being incubated with diluted peroxidase-conjugated secondary antibodies (1:10,000 in 1X TBS-T) at room temperature for 1–2 h. The blots were exposed using the Clarity ECL kit (BioRad, 1,705,061) and imaged on a BioRad ChemiDoc gel imaging system. If needed, membranes were stripped with Restore Western Blot Stripping Buffer (ThermoFisher, 21,059) for 5 m at room temperature. Membranes were washed 3 times for 5 min with 1X TBS-T before being blocked and probed with a new antibody.

Densitometry analysis for pCHK1 was performed by measuring the intensity of each individual pCHK1 band on ImageJ and normalizing to the intensity of its corresponding CHK1 band. The highest ratio between bands of pCHK1 to CHK1 was then normalized to 1 as highest normalized relative intensity.

### Short-term cell viability and drug synergy assays

Cells were trypsinized with 0.05% Trypsin EDTA, counted with 0.4% Trypan blue solution (Gibco), and then seeded at 1,000 cells per well in a 96-well plate in a volume of 100 μL of complete media in triplicate. The following day, the cells in the inner-60 wells of the 96-well plate were treated with various concentrations of drugs as indicated. After drug treatment of 3 days, or 6 days if TMZ treatment was involved, media was flicked off the cells gently. Cells were washed once in 1X PBS, fixed in 4% paraformaldehyde in 1X PBS for 15 min at room temperature, and stained with Hoescht at 1 µg/mL in 1X PBS for 30 min at room temperature. Hoescht was removed from cells and 50 μL of 1X PBS was added to each well in the plates. Plates were imaged on a Cytation 3 (BioTek) and counted using CellProfiler 4.2.1 (http://cellprofiler.org). For synergy assays, synergy was calculated using the software Combenefit^44^.

### Clonogenic survival assay

Cells were pretreated with the indicated concentrations of TMZ in culture for 72 h in 10 cm tissue culture dishes. Cells were then trypsinized with 0.05% Trypsin EDTA, counted with 0.4% Trypan blue solution, and seeded at the proper dilutions in fresh media without drug. Cells were seeded in 6-well plates in triplicate wells, with a threefold dilution ranging from 9000 to 37 cells per well. Immediately after seeding, cells were treated with indicated concentrations of ATR inhibitor BAY-185344 and plates were placed in the 37C incubator. After 12–14 days, media was removed from the cells, washed gently with 1X PBS, and stained with 0.5% crystal violet in methanol for 1 h at room temperature. Crystal violet was carefully poured off the plates and plates were cleaned in water and left to dry overnight at room temperature. Colonies were counted by hand and counts were normalized to the plating efficiency of corresponding drug treatment condition. Where the drug treatment killed all the cells, we counted “0.” We are unable to plot 0 on the logarithmic y-axis of these graphs since log(0) is undefined, so the seemingly missing data point is due to total cell kill from drug treatment.

### Immunofluorescence

Cells were trypsinized with 0.05% Trypsin EDTA, counted with 0.4% Trypan blue solution (Gibco), and then seeded at 10,000 cells/well in clear bottom, black 96-well plates (Grenier, #655866) and placed in the incubator overnight. At the stated times, cells were treated with 100 μM of temozolomide. The cells were fixed and stained as previously described in the pRPA32 S33 protocol^35^. Cells were incubated with primary antibodies at 4C overnight, at 1:1000 dilution of pRPA32 S33 (Bethyl, A300-246A) and 1:500 dilution of anti-phospho-histone H2A.X Ser139 clone JBW301 (Millipore Sigma, 05–636). Secondary antibodies dilutions were 1:500, and Hoescht 33,342 at 1 µg/mL in Blocker Casein in PBS (ThermoFisher). Secondary and Hoescht were removed from the cells and 50 μL of 1X PBS was added to each well in the plates before sealing with an aluminum plate seal. Cells were imaged on a Keyence BZ-X800 and foci were analyzed using the Focinator^45^.

### Flow cytometry

For propidium iodide (PI) staining, cells were seeded in 6-well dishes 24-48 h before drug treatment. After drug treatment at the indicated times, cell media was collected and cells were harvested by trypsinization. The pellet was washed resuspended in 100 μL of 1X PBS on ice. The pellet was fixed with 70% ice-cold ethanol, which was added dropwise to the pellet while gently vortexing. After 30 min of fixing on ice, the samples were centrifuged and ethanol was removed. Then cell pellets were resuspended and stained with RNAse/PI buffer (BD Biosciences, 550,825) 30 min in the dark at room temperature before analysis on a CytoFLEX Flow Cytometer. All experiments were performed in triplicate and analyzed using FlowJo software.

### Statistical analysis

Data are presented as mean ± SD or SEM. Statistical analyses were carried out using GraphPad PRISM 7. Clonogenic survival assays were analyzed using a two-tailed unpaired *t*-test. Flow cytometry data was analyzed using a one-way ANOVA with multiple comparisons. Asterisks indicate levels of significance and *p*-value (* < 0.05, ** < 0.01, *** < 0.001, **** < 0.0001).

## Supplementary Information

Supplementary Information.
