# Supplementary material for: Mismatch repair proteins play a role in ATR activation upon temozolomide treatment in MGMT-methylated glioblastoma
Source: Sci Rep. 2022 Apr 6;12:5827. doi: 10.1038/s41598-022-09614-x (PMC8987098; doi:10.1038/s41598-022-09614-x)

**Mismatch repair proteins play a role in ATR activation upon temozolomide treatment in *MGMT*-methylated glioblastoma**

Sachita Ganesa^1^, Amrita Sule^2^, Ranjini K. Sundaram^2^, Ranjit S. Bindra^2^

^1^ Department of Molecular Biophysics and Biochemistry, Yale University, New Haven, CT 06511

^2^ Department of Therapeutic Radiology, Yale University, New Haven, CT 06511

Corresponding author:

Ranjit S. Bindra

Yale School of Medicine

333 Cedar Street, New Haven, CT 06520

Phone: 203-200-3672

Fax: 203-200-3673

Email: [ranjit.bindra@yale.edu](mailto:ranjit.bindra@yale.edu)

# **Supplementary Figures**


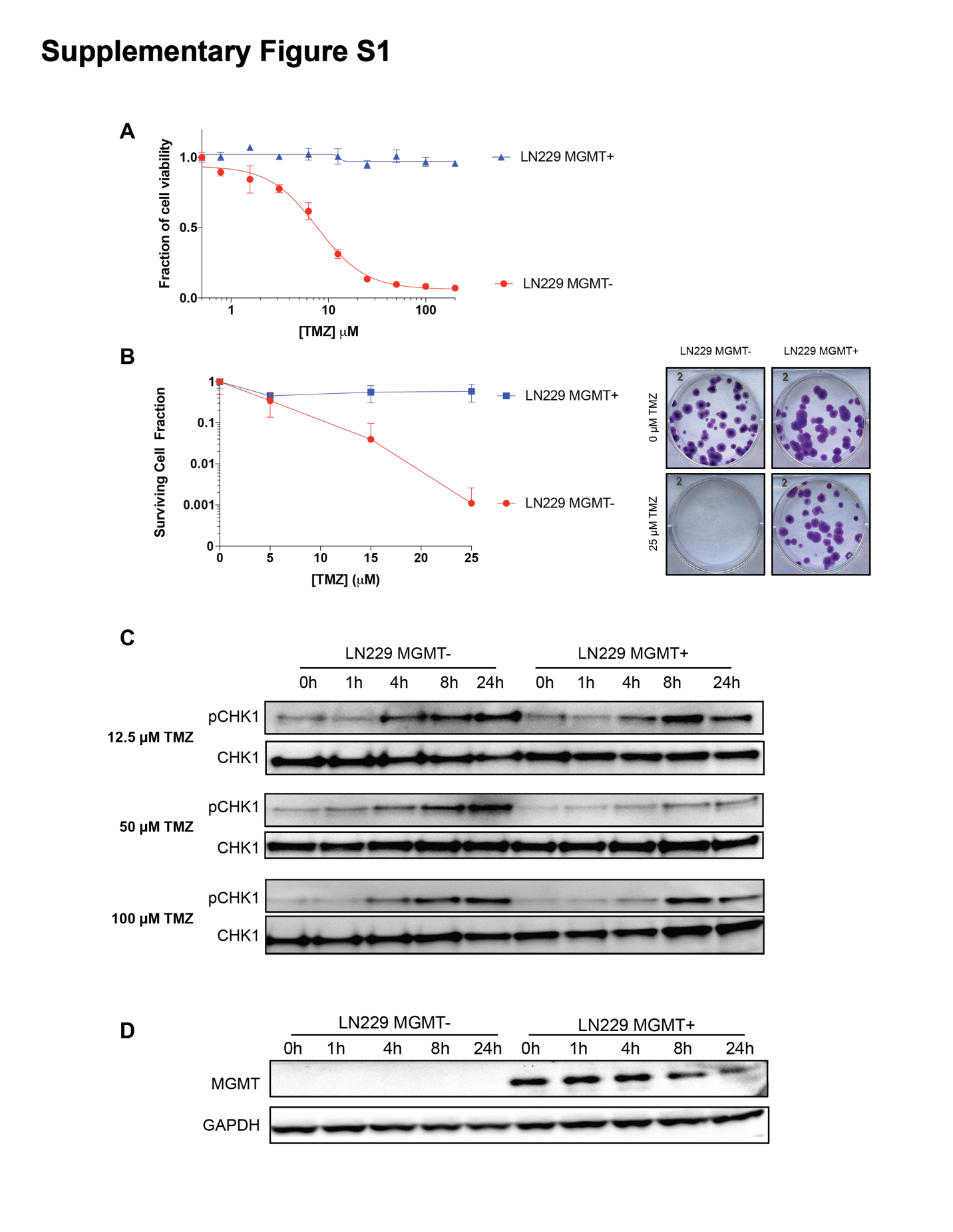


-51 kDa

-51 kDa

-51 kDa

-51 kDa

-51 kDa

-51 kDa

-21 kDa

-37 kDa

**Supplementary Figure S1. MGMT status affects ATR activation upon TMZ treatment.** (A) LN229 MGMT- cells are sensitive to TMZ in short-term cell viability assay compared to MGMT+ cells. Cells were treated in triplicate (*n*=3) for 6 days with temozolomide before being fixed, stained, and quantified. (B) Clonogenic survival assay reiterates that LN229 MGMT- cells are sensitive to TMZ compared to MGMT+ cells which are resistant. Cells were pre-treated with temozolomide for 72 hours before being seeded in fresh media. Cell colonies incubated for 14 days before being fixed, stained, and quantified. The crystal violet staining of the colonies is seen upon various drug treatments. (C) Immunoprecipitation of CHK1 shows an increase in pCHK1 levels earlier in MGMT- cells than MGMT+ cells indicating increased ATR activation. Cells were treated with the indicated concentrations of temozolomide for the indicated time points before being pelleted and lysed for immunoprecipitation with a CHK1 pull-down. (D) Representative western-blot of the whole cell lysate fractions from the 12.5 μM TMZ concentration showing MGMT status. Full length blots shown below. Blots were cut prior to hybridization with primary antibody.


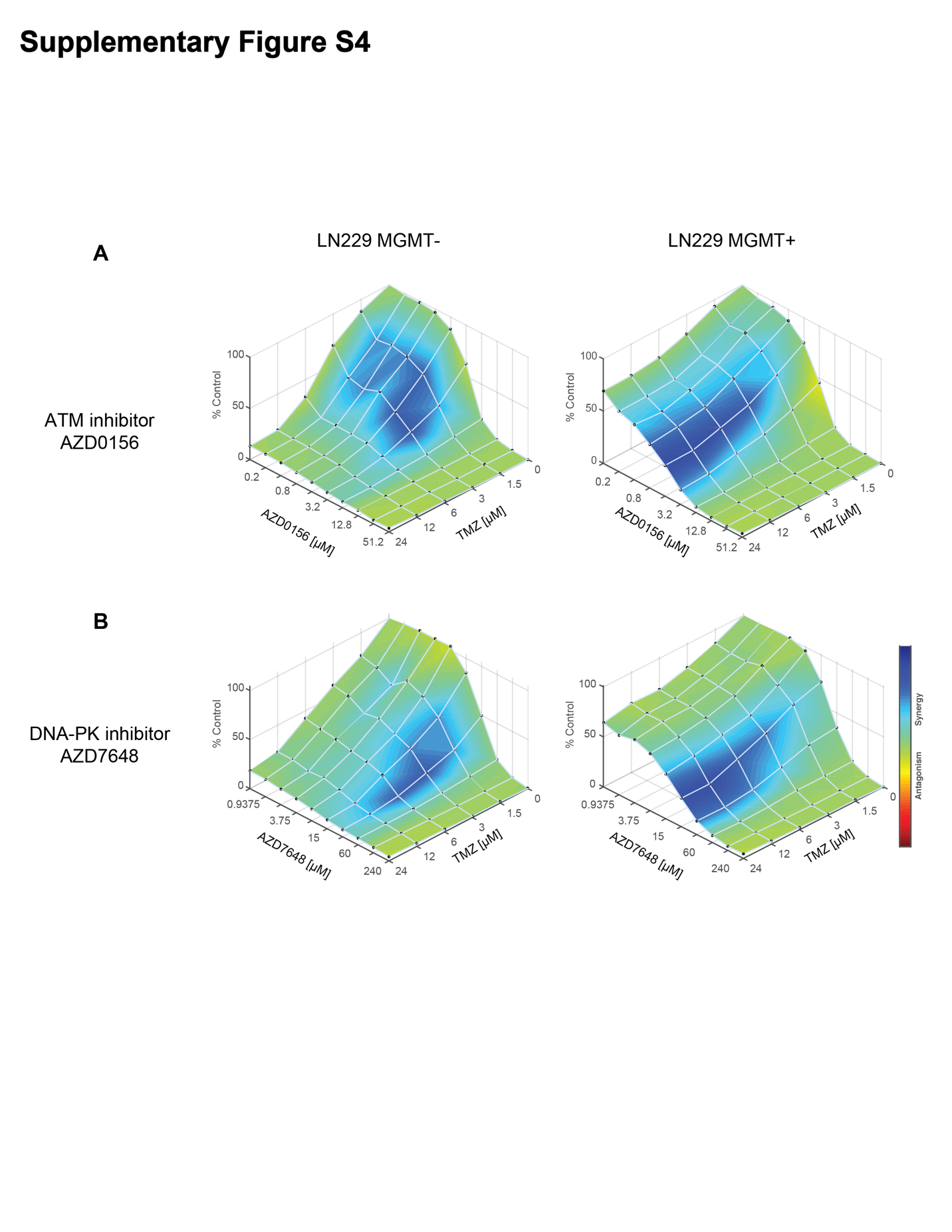


**Supplementary Figure S2. MMR-induced synergy is unique to TMZ and ATR inhibitors**. MGMT- cells and MGMT+ cells exhibit synergy with TMZ in combination with (A) ATM inhibitor AZD0156, or with (B) DNA-PK inhibitor AZD7648, suggesting that MGMT-status only plays a role in the synergy observed between TMZ and ATR inhibitor. LN229 cells were seeded in triplicate (*n*=3) and were treated with the combination of drugs for 6 days before being fixed, stained, and quantified. Drug synergy analysis was completed using the Combenefit software.

**
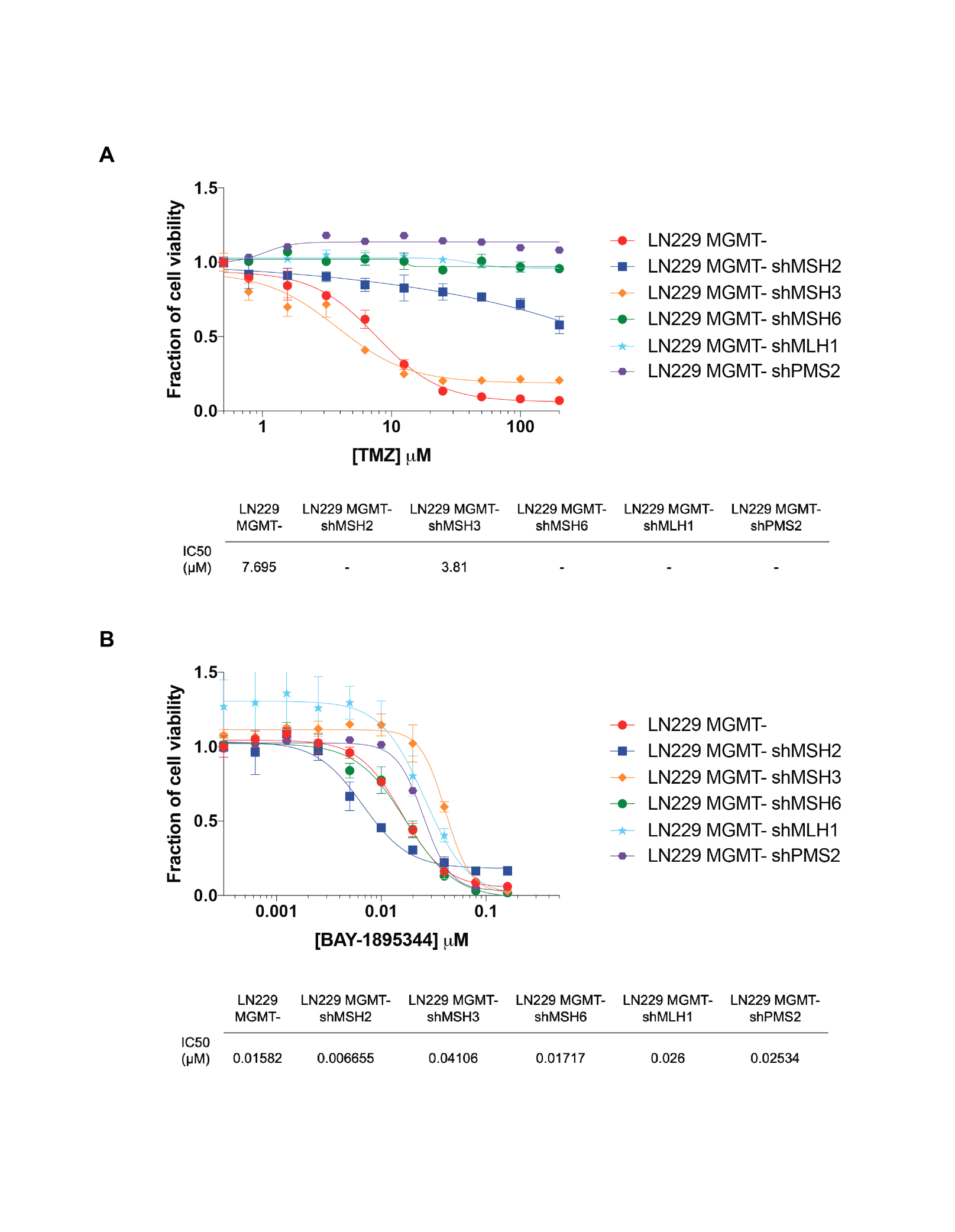
**

**Supplementary Figure S3. Sensitivity of MMR cells to TMZ and ATR inhibitor BAY-1895344.** LN229 cells were treated in triplicate (*n*=3) with TMZ for 6 days or BAY-1895344 for 3 days before being fixed, stained, and quantified using CellProfiler. (A) Short-term cell viability assays in all LN229 MGMT- cell lines, both MMR-proficient and MMR-deficient with TMZ and (B) ATR inhibitor BAY-1895344. shMSH2, shMSH6, shMLH1, and shPMS2 cells are resistant to TMZ, whereas shMSH3 is sensitive. All shMMR cells are equally sensitive to BAY-1895344.

**
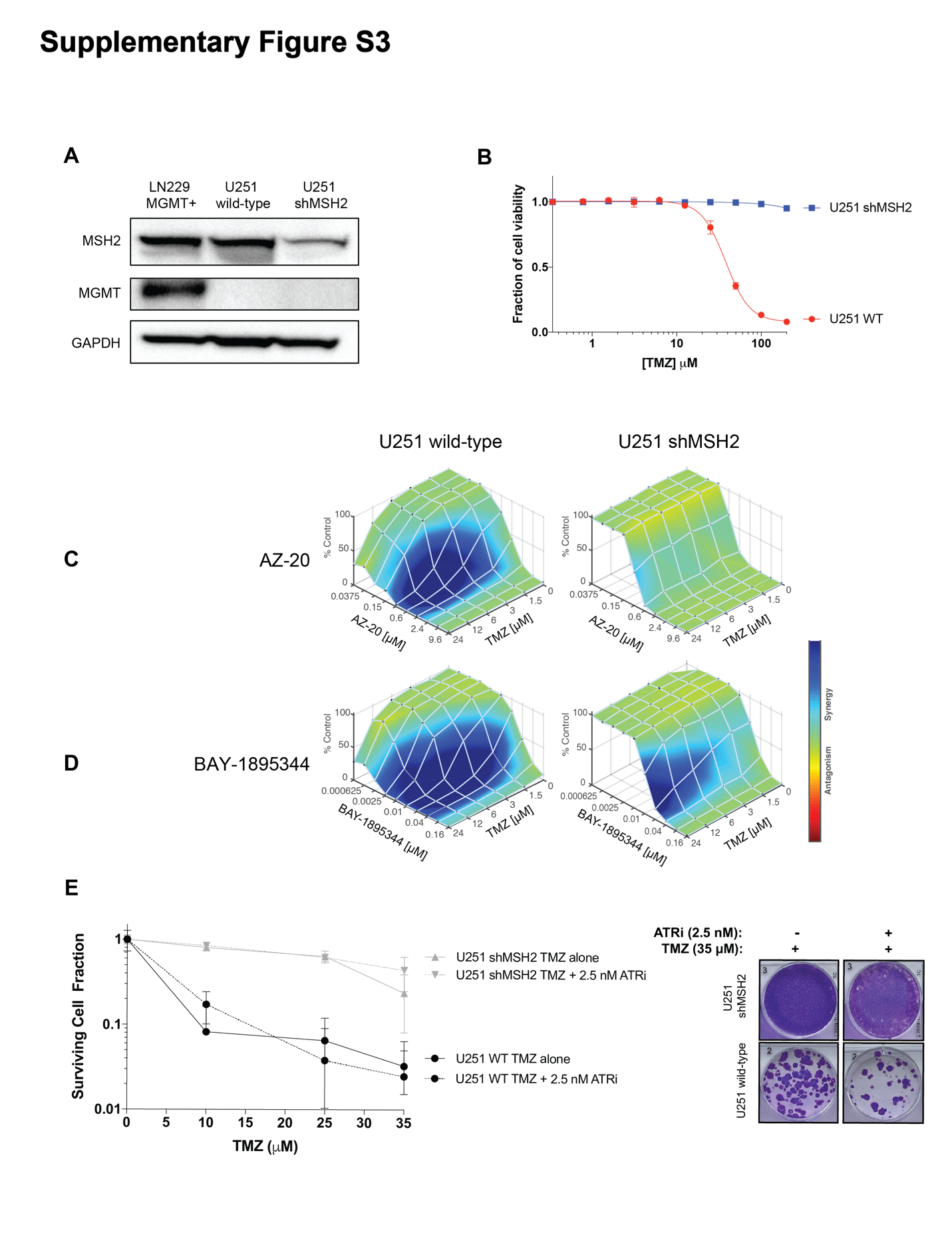
**

**Supplementary Figure S4. U251 shMSH2 glioblastoma cells display same phenotype as LN229 shMSH2 cells.** (A) Western blot showing MSH2 and MGMT status of U251 cells compared to LN229 MGMT+ cells. Full length blots shown below. Blots were cut prior to hybridization with primary antibody. (B) U251 wild-type cells are sensitive to TMZ as a monotherapy unlike U251 shMSH2 cells in short-term cell viability assay, similarly to the LN229 cells that are MSH2-proficient and MSH2-deficient. U251 cells were treated in triplicate (*n*=3) with temozolomide for 6 days before being fixed, stained, and quantified with CellProfiler. (C) U251 shMSH2 cells exhibit an abrogation of synergy compared to U251 wild-type cells upon treatment with TMZ and ATR inhibitor AZ-20 and, (D) BAY-1895344. U251 cells were plated in triplicate (*n*=3) and treated with the combination therapy for 6 days before being fixed, stained, and quantified. Drug synergy was analyzed using Combenefit. (E) U251 shMSH2 cells are resistant to TMZ and the combination of TMZ and ATR inhibitor in clonogenic survival assay. Cells were pre-treated with temozolomide for 72 hours before seeding in triplicate (*n*=3) into fresh media and adding BAY-1895344 where indicated. Cells were incubated for 14 days before being fixed and stained with crystal violet. Representative colony images are shown.

**Supplementary – Uncropped blots**

Figure 1A.


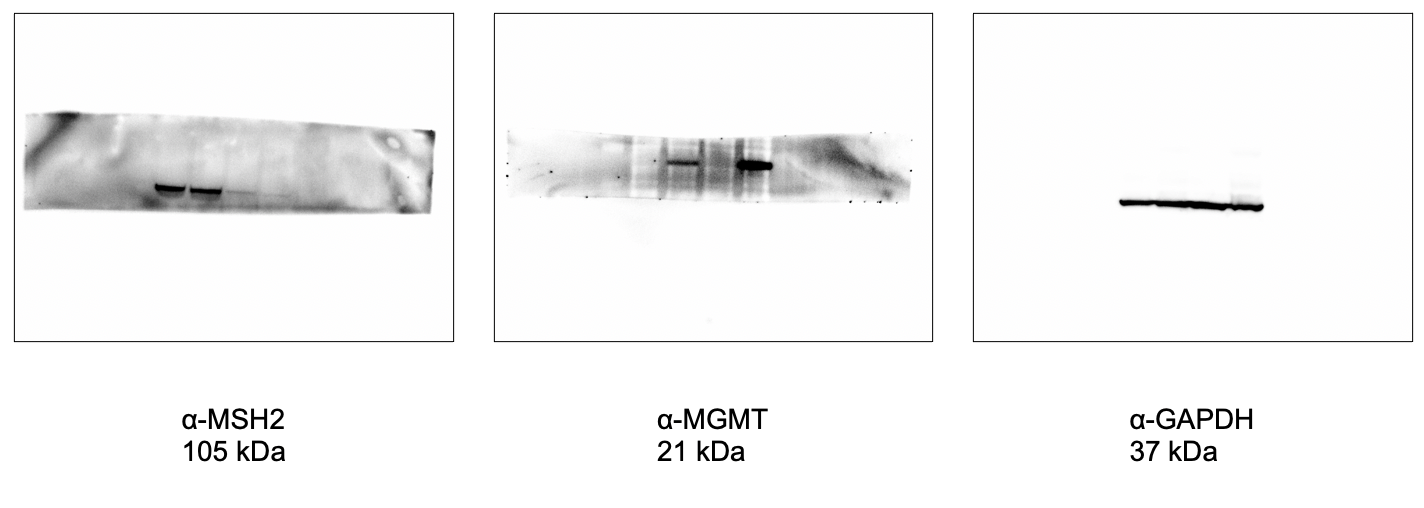


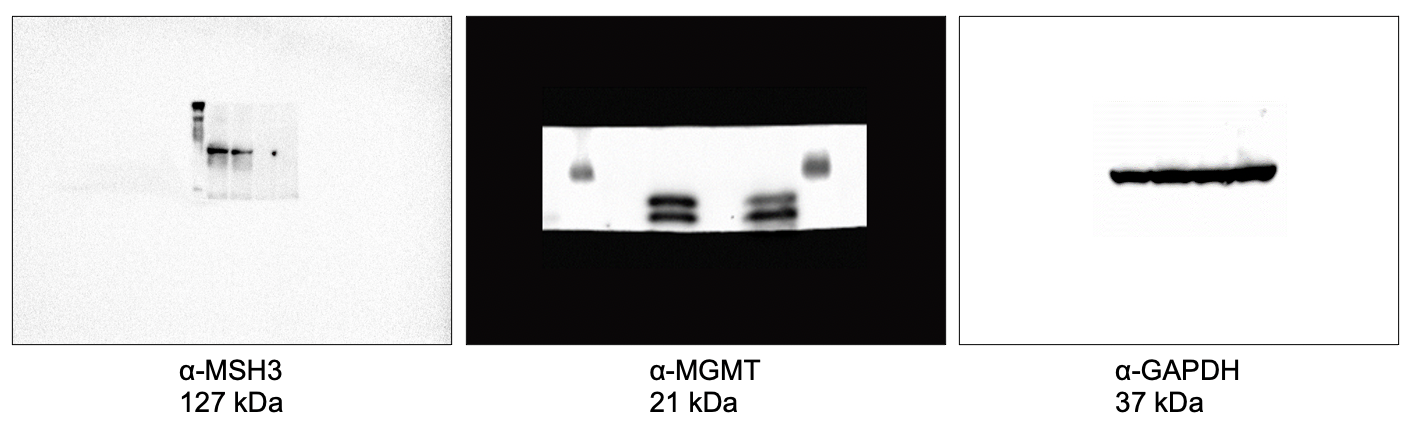


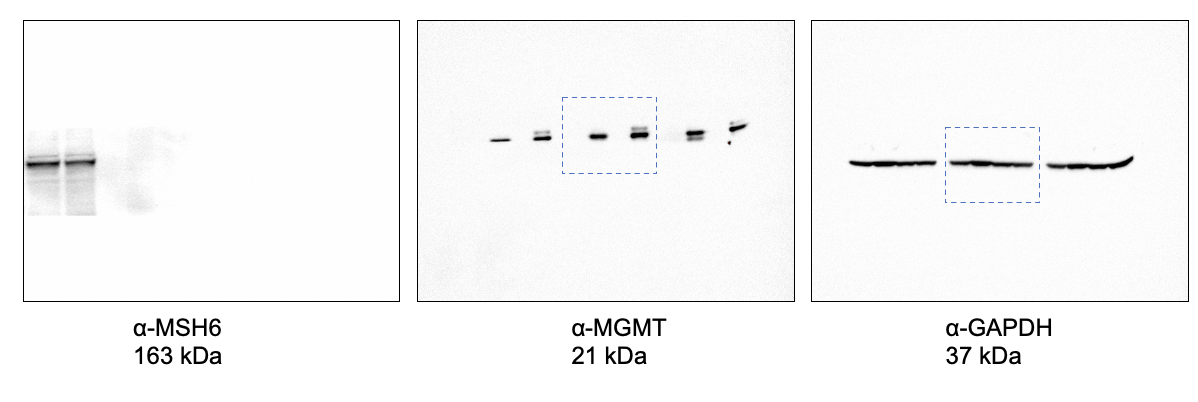


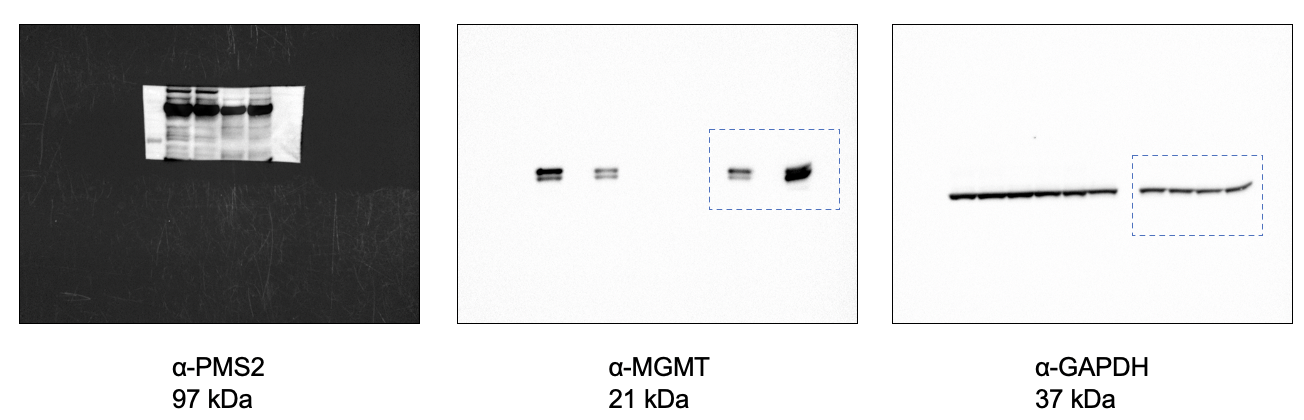


Figure 1B.


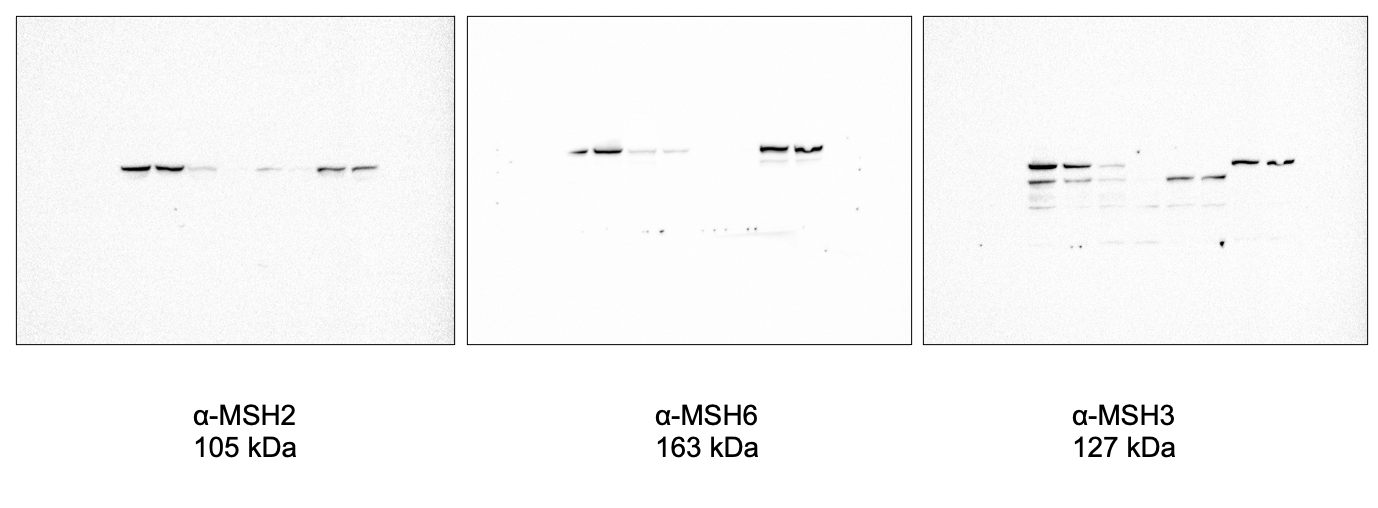


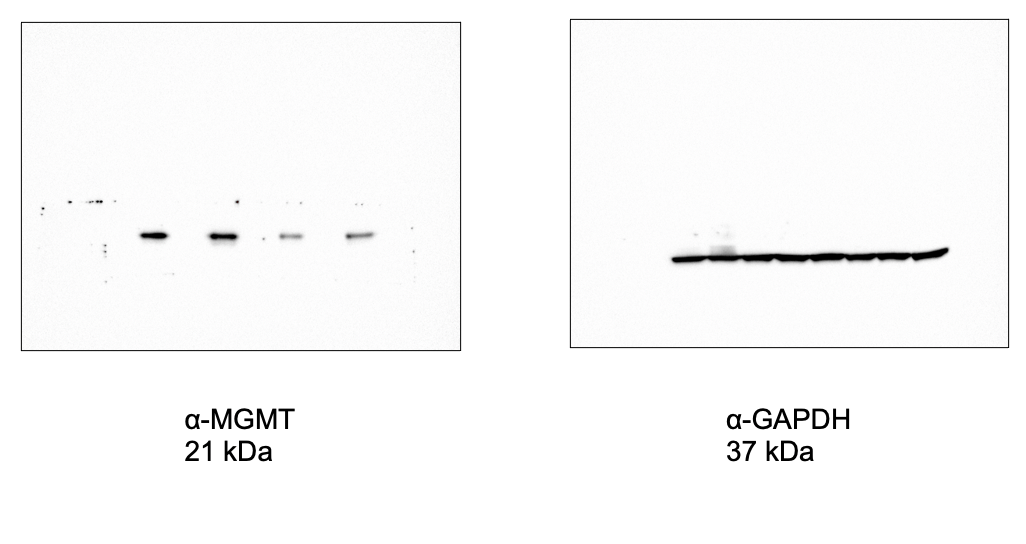


Figure 1A/C.


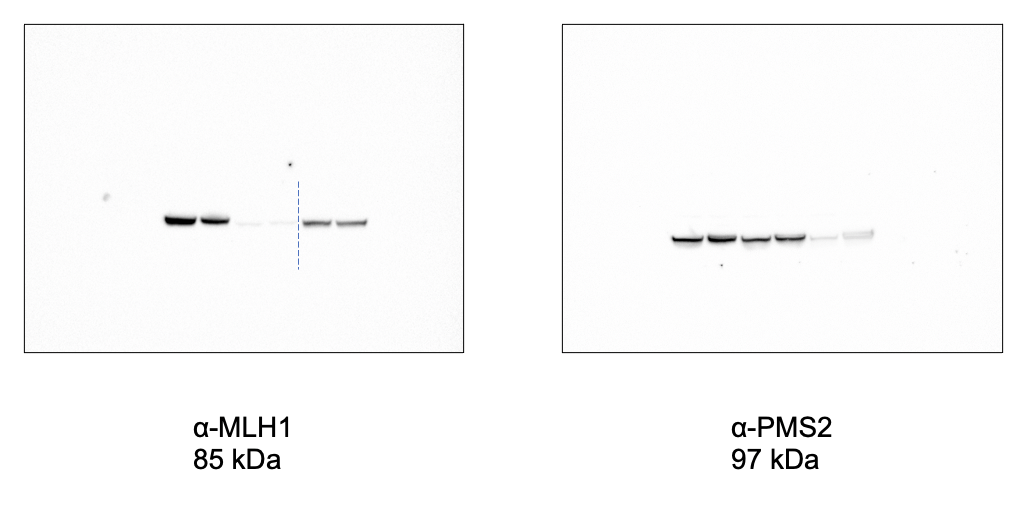


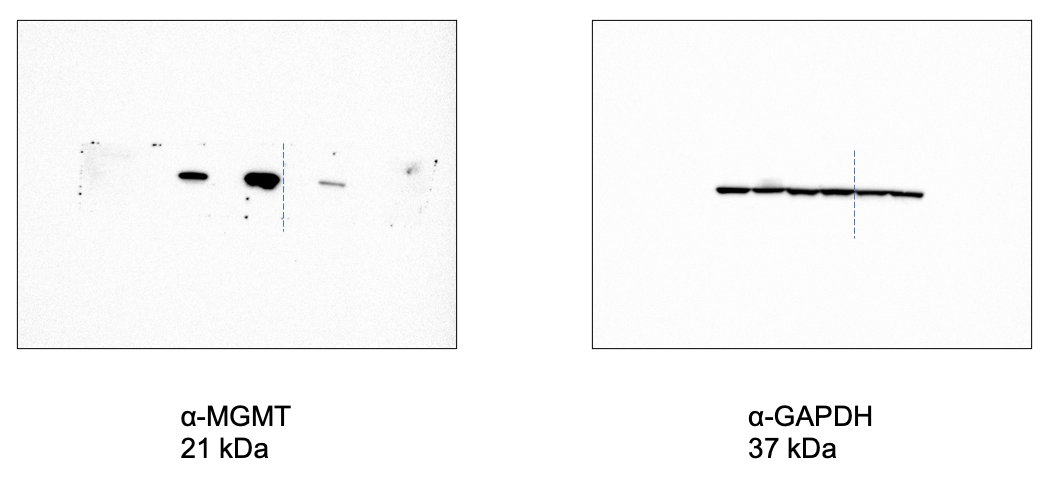


Figure 4G.


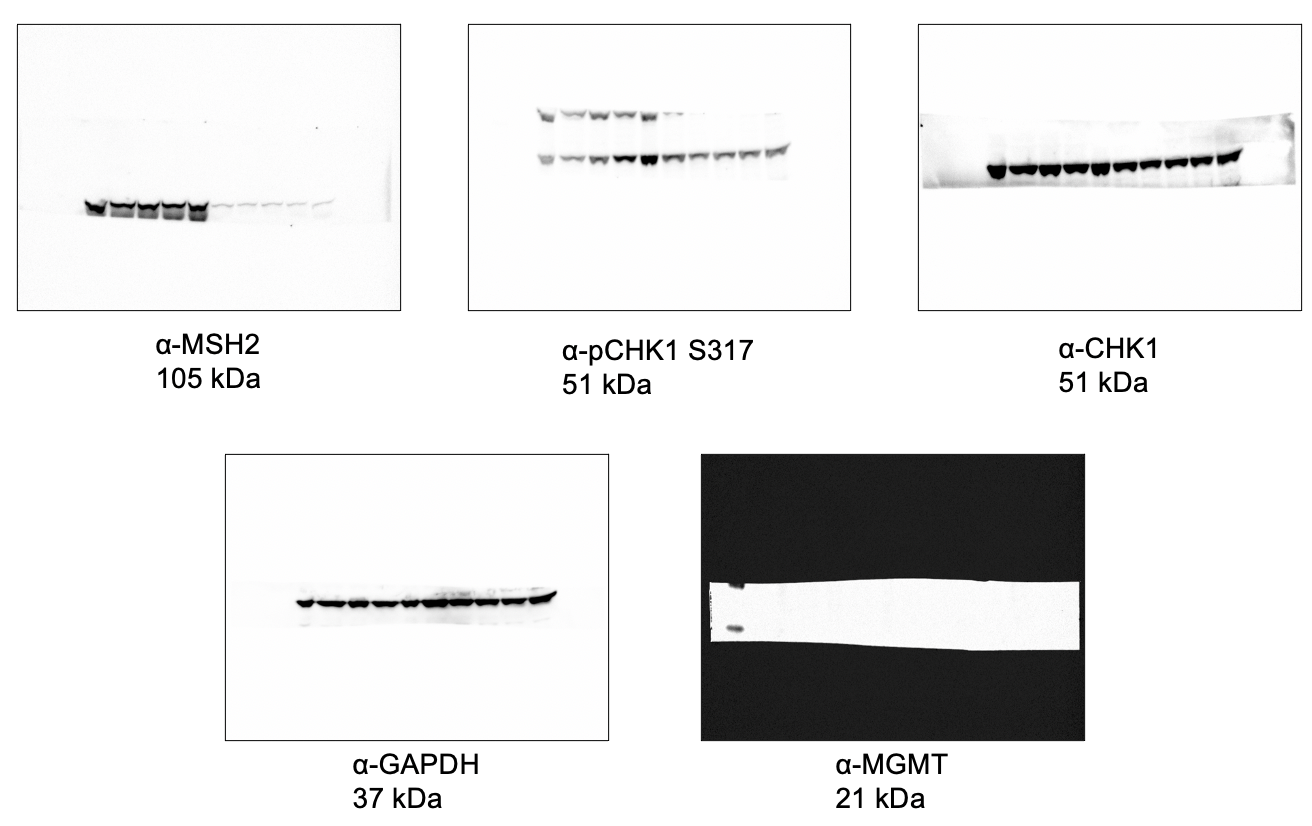


Supplementary Figure 1C and D.


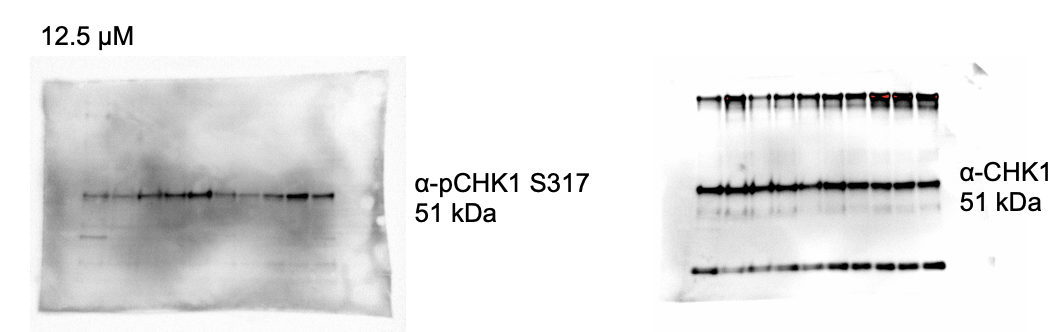


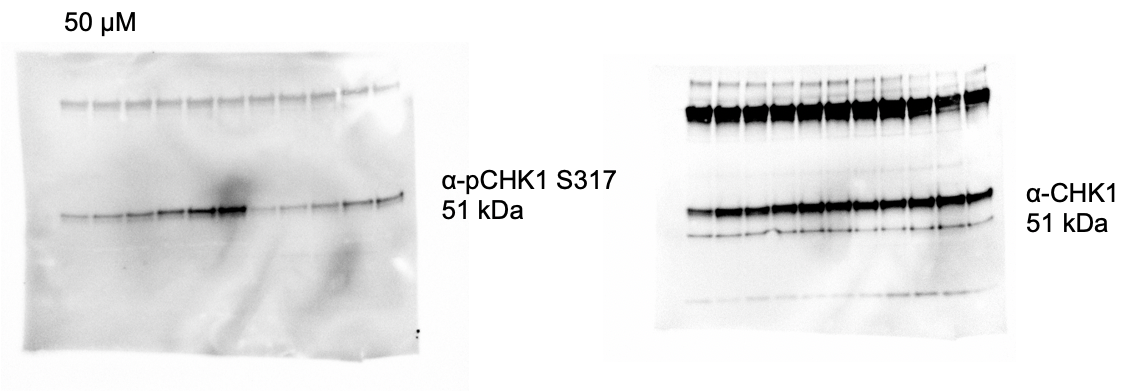


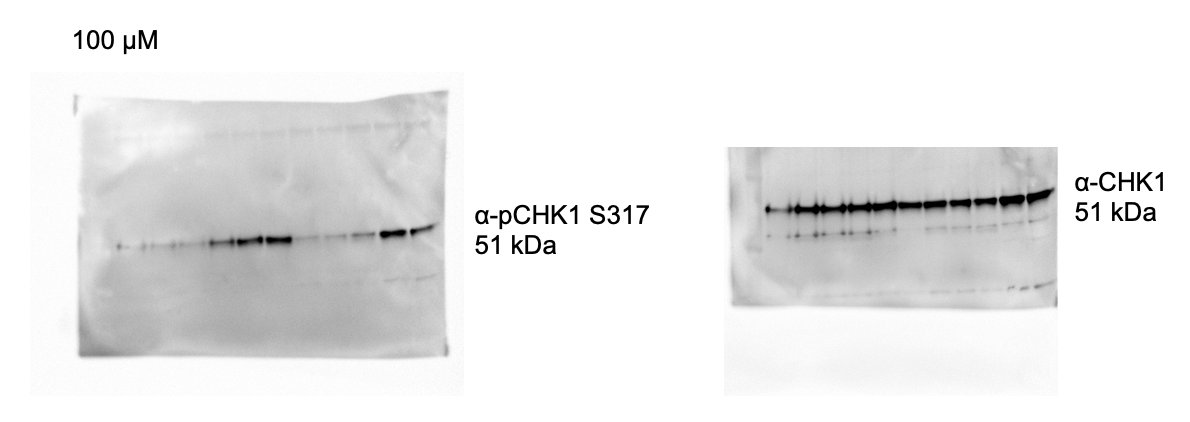


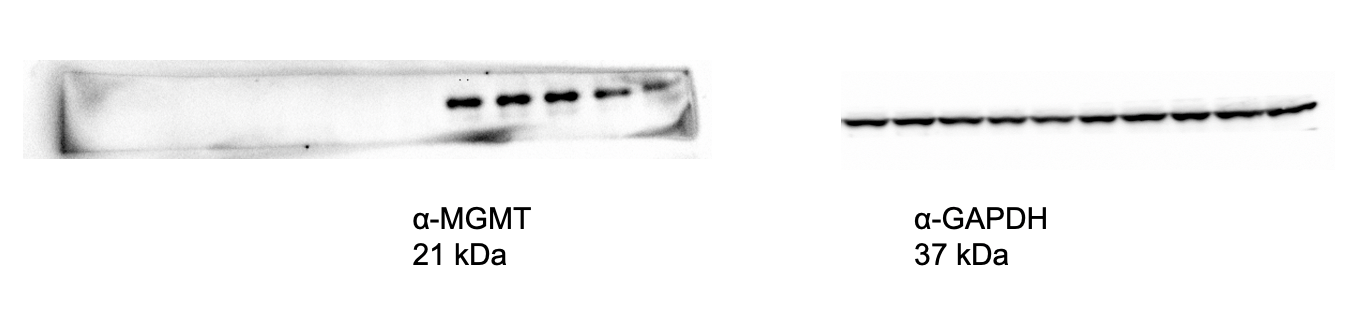


Supplementary Figure 4A.


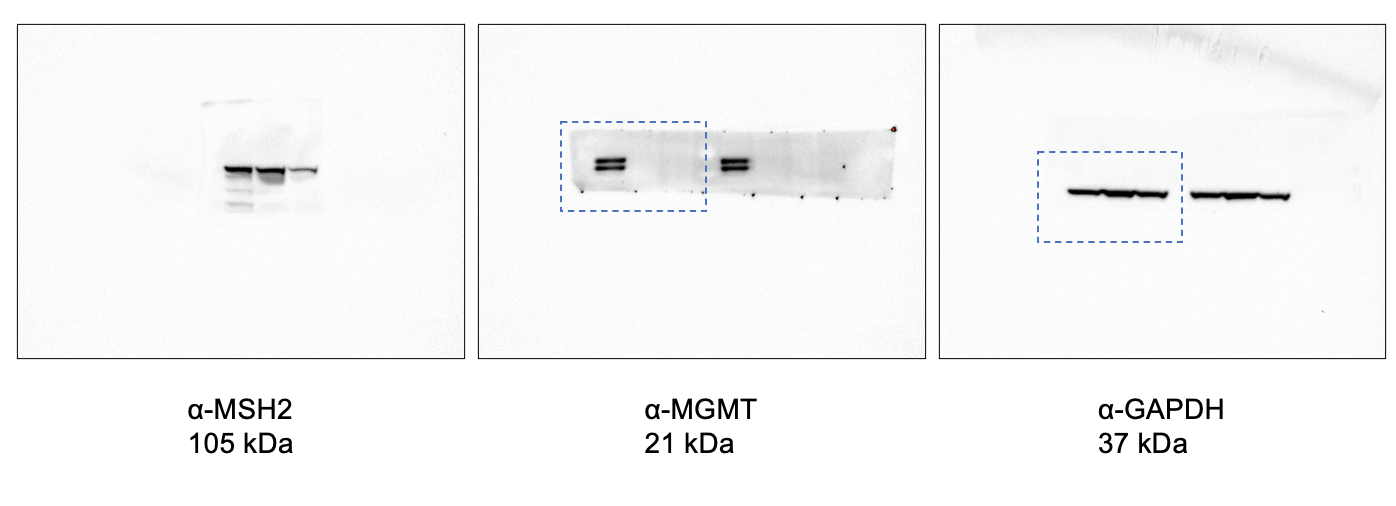

Supplement: Supplementary file 1 — Supplementary Information. [file 41598_2022_9614_MOESM1_ESM.docx]
